# Supplementary figures and images for: Single-Cell Genomics Reveals the Divergent Mitochondrial Genomes of Retaria (Foraminifera and Radiolaria)
Source: mBio. 2023 Mar 20;14(2):e00302-23. doi: 10.1128/mbio.00302-23 (PMC10127745; doi:10.1128/mbio.00302-23)

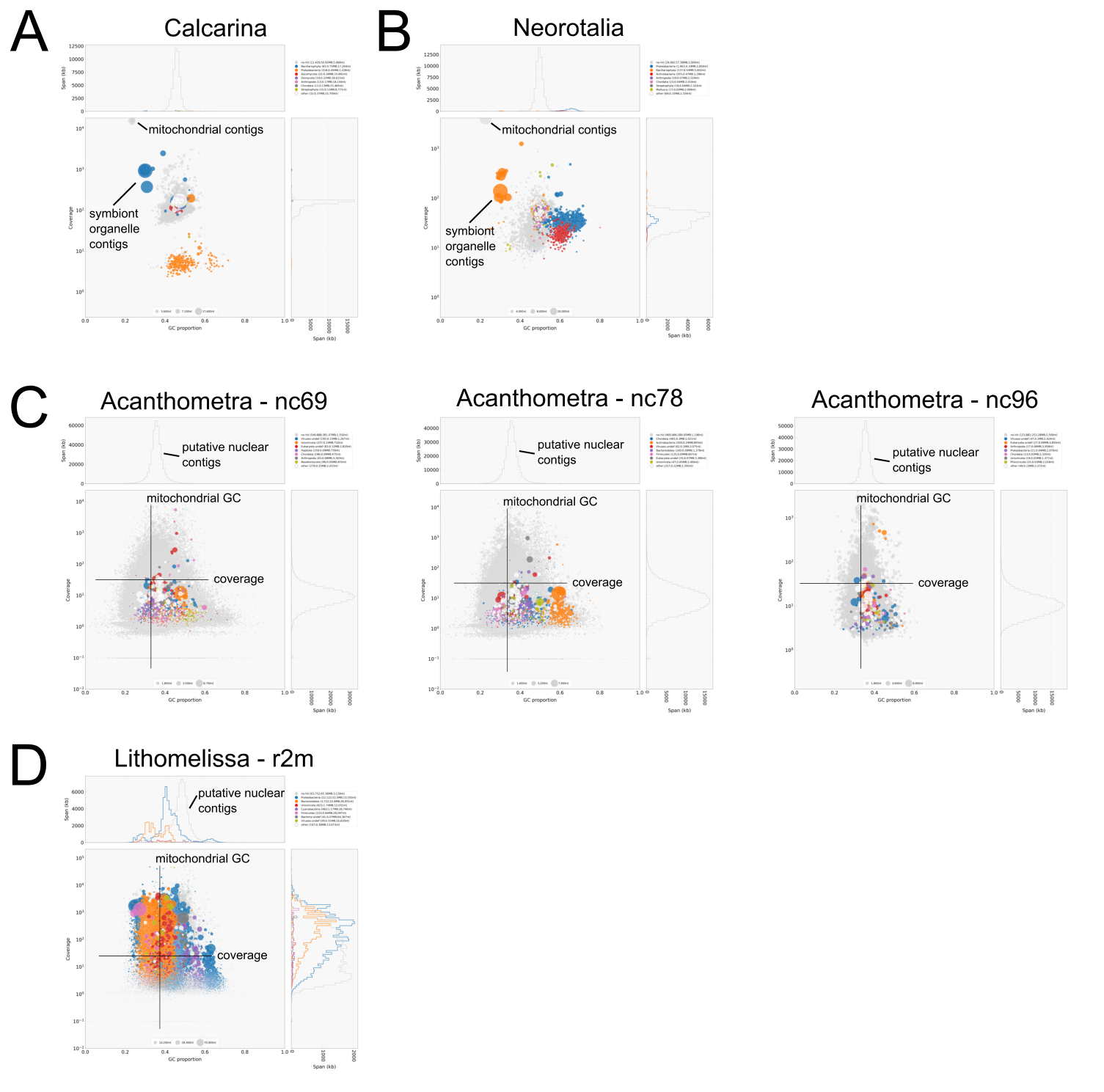

Supplement: FIG S1 [file mbio.00302-23-s002.tif]

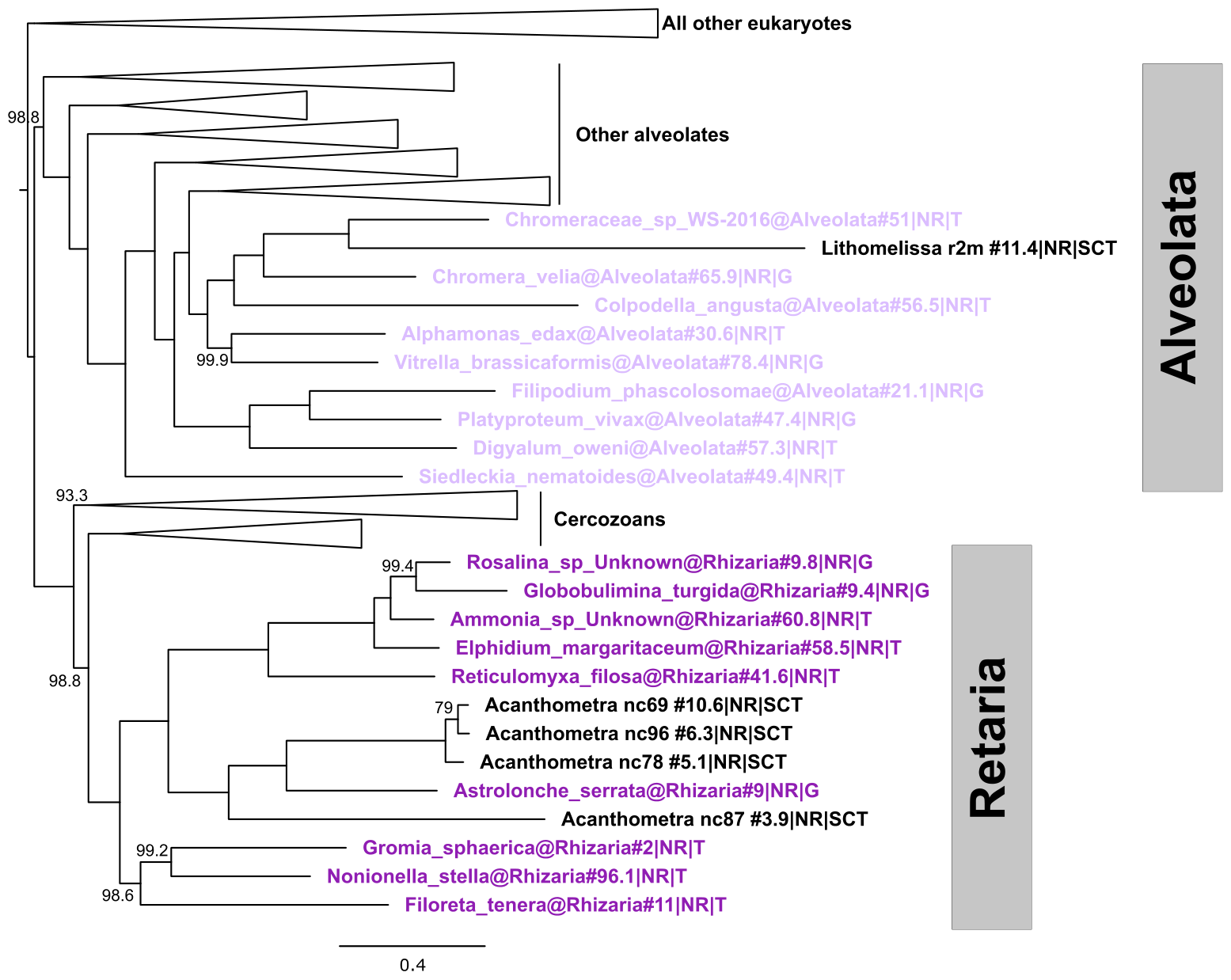

Supplement: FIG S2 [file mbio.00302-23-s004.tif]

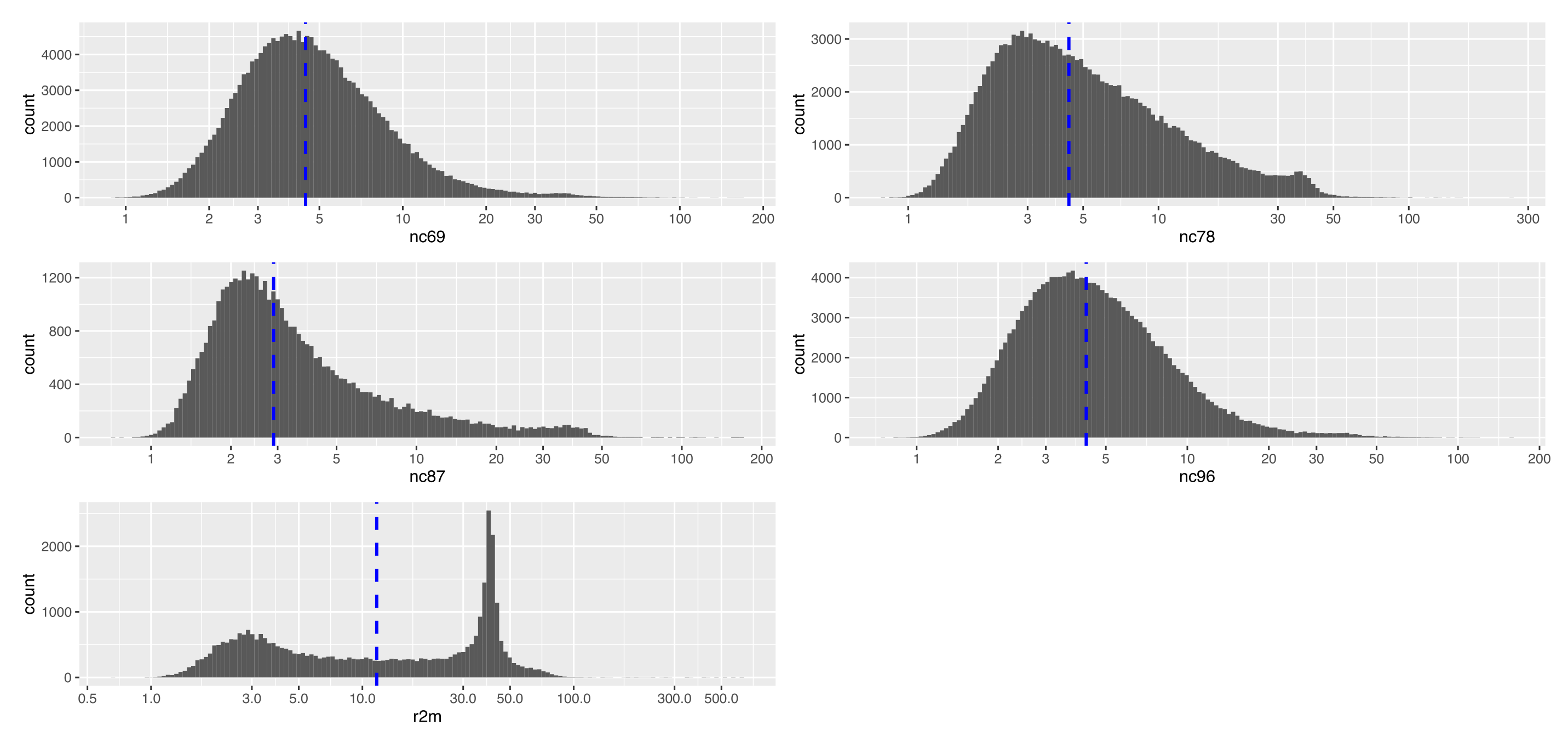

Supplement: FIG S3 [file mbio.00302-23-s005.tif]

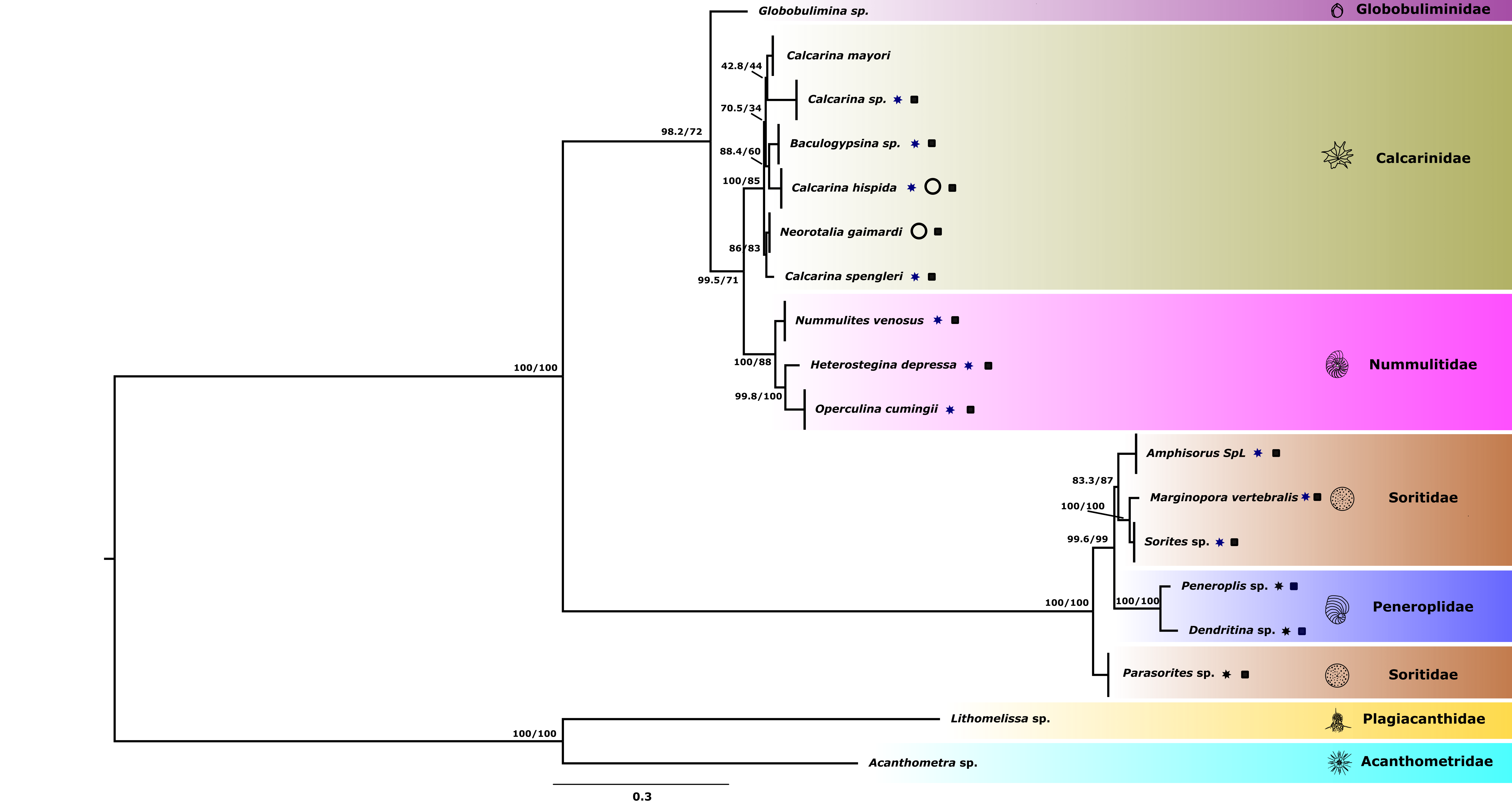

Supplement: FIG S4 [file mbio.00302-23-s006.tif]

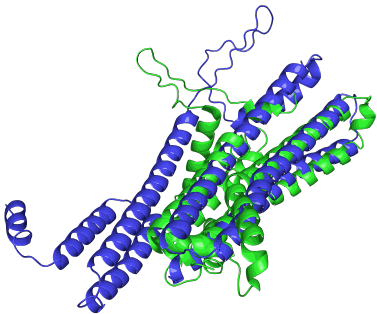

Supplement: FIG S5 [file mbio.00302-23-s007.pdf]
